# Asymptomatic endobronchial tuberculosis represented as a solitary airway stenosis without tree‐in‐bud appearance on computed tomography

**DOI:** 10.1002/ccr3.4742

**Published:** 2021-09-24

**Authors:** Hirokazu Toyoshima, Motoaki Tanigawa

**Affiliations:** ^1^ Department of Infectious Diseases Japanese Red Cross Ise Hospital Ise Japan; ^2^ Department of Respiratory Medicine Japanese Red Cross Ise Hospital Ise Japan

**Keywords:** asymptomatic, bronchoscopy, endobronchial tuberculosis, high‐resolution computed tomography, solitary airway stenosis

## Abstract

Bronchoscopy is a crucial tool for diagnosing endobronchial tuberculosis in patients with airway stenosis. Early diagnosis and treatment may reduce airway sequelae and prevent the spread of infection.

## Graphical abstract

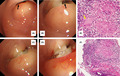

## 1 CLINICAL IMAGE

Patients with endobronchial tuberculosis can be asymptomatic despite its high contagiousness. High‐resolution computed tomography can reveal solitary stenosis in the peripheral bronchus without tree‐in‐bud appearance. However, in such patients, targeted bronchial lavage and brushing, fluid sampling, and biopsy, followed by aggressive bronchoscopy, may prevent airway sequelae with appropriate antituberculosis medicine.

**FIGURE 1:**
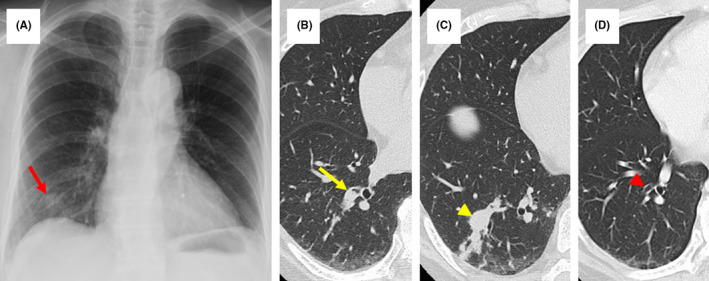
Imaging findings. Chest X‐ray findings showing a 15 × 16‐mm‐sized nodule in the right lower lobe of the lung (A, red arrow). Chest computed tomography findings showing stenosis in the right B9 bronchus (B, yellow arrow) and a 13 × 16‐mm‐sized elliptic nodule with consolidation in the S9 segment of the right lower lobe (C, yellow arrowhead). No visible stenosis in the B9 bronchus at 2 years after the first‐line antituberculosis administration is observed (D, red arrowhead)

**FIGURE 2:**
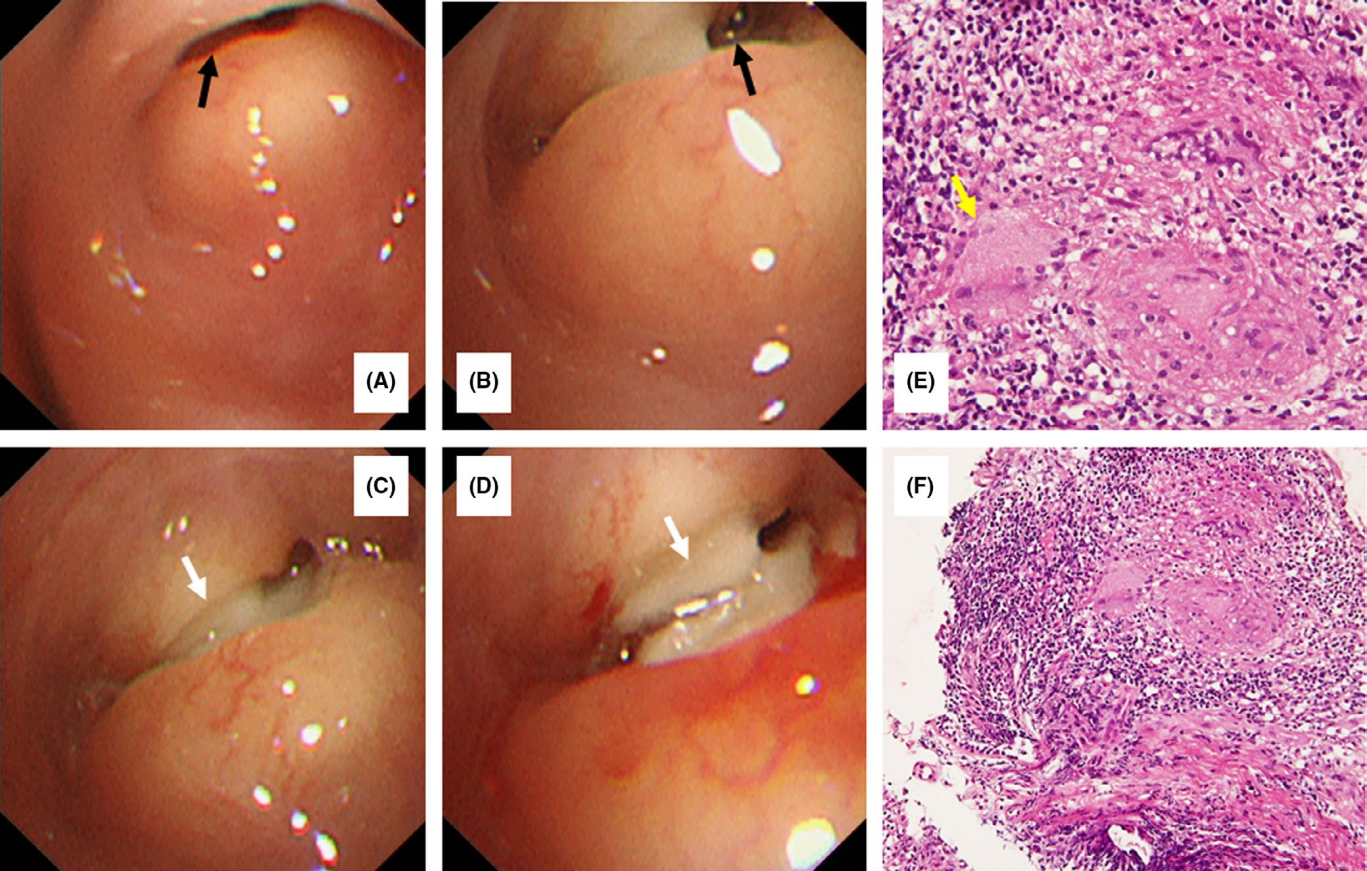
Pathology of the endobronchial lesions. Bronchoscopy findings showing a circular elevated endobronchial lesion with stenosis (A and B, black arrows) and ulceration (C and D, white arrows) in the orifice of the right B9 bronchus. The surrounding mucosa appears normal. Histology of the endobronchial lesion showing granulation with multinucleated giant cells (hematoxylin and eosin staining, original magnification ×10) (E, yellow arrow), without caseous necrosis (hematoxylin and eosin staining, original magnification ×400) (F)

A 65‐year‐old Japanese woman with cervical cancer history was examined annually for 11 years without recurrence. Chest computed tomography (CT) during a routine follow‐up examination showed a right S9 pulmonary segment nodule with right B9 bronchus stenosis (Figure 1). She was asymptomatic. Bronchoscopy revealed a circular elevated lesion and ulceration with stenosis in the right B9 bronchus orifice (Figure 2). Bronchial lavage fluid, bronchial brushing, and ulcer biopsy samples were collected. The acid‐fast staining and the polymerase chain reaction findings were negative. However, histology suggested tuberculosis. Subsequently, an acid‐fast bacilli culture of the bronchial lavage fluid and bronchial brushings yielded a positive result for *Mycobacterium tuberculosis*. She was diagnosed with endobronchial tuberculosis (EBTB) and received a 6‐month first‐line antituberculosis antibiotic treatment. She had no recurrence or airway sequelae during the 2‐year follow‐up period (Figure 1).

Limited asymptomatic patients with EBTB can be incidentally diagnosed during an annual checkup. High‐resolution CT may show solitary airway stenosis without tree‐in‐bud appearance. Bronchoscopy is recommended, as bronchial lavage fluid and brushings yield positive culture results more frequently than sputum.^1^ Bronchial stenosis may develop in 60%–95% of cases despite adequate antituberculosis antibiotic administration.^2^ Rapid diagnosis and early first‐line antituberculosis antibiotic administration contribute to disappearance without recurrence.

## CONFLICT OF INTEREST

There are no conflicts of interest to declare.

## AUTHOR CONTRIBUTIONS

HT: Clinical management of the patient, study conception, acquisition, and analysis of data, manuscript draft. TM: Supervision of the drafting of the manuscript and critical revision of the manuscript. All authors: Review of the final draft of the manuscript and approval of the manuscript's submission.

## ETHICAL APPROVAL

This study was approved by the institutional review board and ethics committee of the Japanese Red Cross Ise Hospital (approval number: ER2020‐26).

## CONSENT FOR PUBLICATION

Written informed consent was obtained from the patient for the publication of this case report and the accompanying images.

## Data Availability

The data that support the findings of this study are openly available in [repository name e.g “figshare”] at [http://doi.org/10.1002/ccr3.4742], reference number [CCR3‐2021‐05‐0882‐IV.R1].

## References

[1] Lee JH, Park SS, Lee DH, Shin DH, Yang SC, Yoo BM. Endobronchial tuberculosis. Clinical and bronchoscopic features in 121 cases. Chest. 1992;102:990‐994. 1395814 10.1378/chest.102.4.990

[2] Kashyap S, Solanki A. Challenges in endobronchial tuberculosis: from diagnosis to management. Pulm Med. 2014;2014:594806. 25197570 10.1155/2014/594806 PMC4147266

